# Translating the Role of mTOR- and RAS-Associated Signalopathies in Autism Spectrum Disorder: Models, Mechanisms and Treatment

**DOI:** 10.3390/genes12111746

**Published:** 2021-10-30

**Authors:** Verica Vasic, Mattson S. O. Jones, Denise Haslinger, Lisa S. Knaus, Michael J. Schmeisser, Gaia Novarino, Andreas G. Chiocchetti

**Affiliations:** 1 Institute for Microscopic Anatomy and Neurobiology, University Medical Center of the Johannes Gutenberg-University, 55131 Mainz, Germany; vasic@uni-mainz.de (V.V.); mschmeisser@uni-mainz.de (M.J.S.); 2 Autism Therapy and Research Center of Excellence, Department of Child and Adolescent Psychiatry, Psychosomatics and Psychotherapy, University Hospital Frankfurt, 60528 Frankfurt am Main, Germany; mattson.jones@kgu.de (M.S.O.J.); Denise.haslinger@kgu.de (D.H.); 3 Center for Personalized Translational Epilepsy Research (CePTER), Goethe University Frankfurt, 60528 Frankfurt am Main, Germany; 4 Institute of Science and Technology (IST) Austria, 3400 Klosterneuburg, Austria; lisa.knaus@ist.ac.at (L.S.K.); gaia.novarino@ist.ac.at (G.N.); 5 Focus Program Translational Neurosciences (FTN), University Medical Center of the Johannes Gutenberg-University, 55131 Mainz, Germany

**Keywords:** Autism Spectrum Disorder, mTOR, RAS, intellectual disability

## Abstract

Mutations affecting mTOR or RAS signaling underlie defined syndromes (the so-called mTORopathies and RASopathies) with high risk for Autism Spectrum Disorder (ASD). These syndromes show a broad variety of somatic phenotypes including cancers, skin abnormalities, heart disease and facial dysmorphisms. Less well studied are the neuropsychiatric symptoms such as ASD. Here, we assess the relevance of these signalopathies in ASD reviewing genetic, human cell model, rodent studies and clinical trials. We conclude that signalopathies have an increased liability for ASD and that, in particular, ASD individuals with dysmorphic features and intellectual disability (ID) have a higher chance for disruptive mutations in RAS- and mTOR-related genes. Studies on rodent and human cell models confirm aberrant neuronal development as the underlying pathology. Human studies further suggest that multiple hits are necessary to induce the respective phenotypes. Recent clinical trials do only report improvements for comorbid conditions such as epilepsy or cancer but not for behavioral aspects. Animal models show that treatment during early development can rescue behavioral phenotypes. Taken together, we suggest investigating the differential roles of mTOR and RAS signaling in both human and rodent models, and to test drug treatment both during and after neuronal development in the available model systems.

## 1. Introduction

The etiology of Autism Spectrum Disorder (ASD) is predominantly genetic with an estimated heritability of 0.80 (0.59–0.95) including rare and common variants that contribute to the phenotypes with varying penetrance [1]. All together many distinct genes have been associated with ASD. However, the convergence of ASD risk genes along specific signaling and regulatory pathways led to the hypothesis that ASD often arises due to defects in neuronal development and signaling. Disruption of discrete signaling cascades often presents with specific phenotypic profiles or syndromes. To this end, the most prominent groups of syndromes associated with ASD are related to disruptions of the mTOR (mechanistic Target of Rapamycin, mTORopathies) and the RAS-RAF-MEK-ERK pathway (RASopathies).

The association of these specific signalopathies with core features of ASD is well founded and based on observations made in patients, animal, and cellular models. However, a translation into clinical practice including refined diagnosis and therapy is still lacking. Here, we review the current knowledge on the syndromes associated with a disruption of the mTOR and RAS-RAF-MEK-ERK pathways and assess the potential impact of these signalopathies with the aim to evaluate their relevance in the clinical setting. 

### 1.1. The mTOR Pathway

Mutations in genes encoding proteins belonging to the mTOR pathway are strongly associated with ASD features. The SFARI gene database (https://gene.sfari.org/, accessed on 1 October 2021), which lists and scores ASD-risk genes based on published literature, categorizes several mTOR pathway-related genes as high risk for ASD. In addition, disruptive variations of these genes are also heavily comorbid with epilepsy, learning disabilities, cancers and other neurodevelopmental disorders creating difficulties in identifying purely ASD-associated genes. 

**Figure 1:**
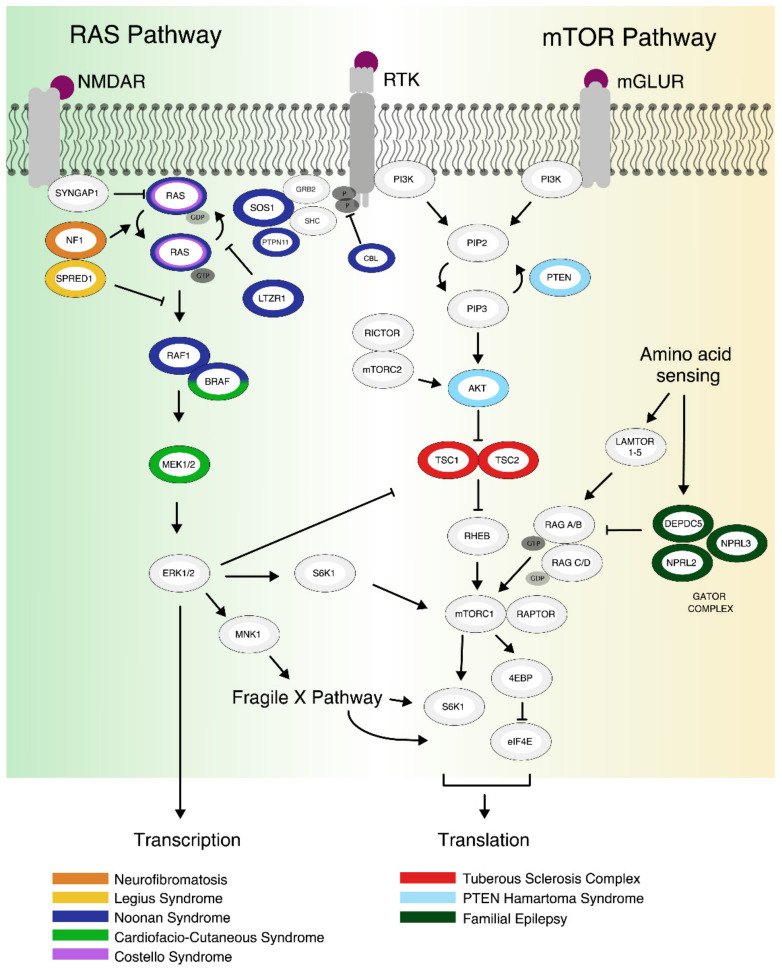
Schematic overview of the RAS and mTOR pathways highlighting clinical syndromes. For abbreviations, see list of abbreviations at the beginning of the article. The figure is based on previous reviews [5] and extended.

At the functional level, the mTOR pathway (Figure 1) maintains and stimulates cell growth and metabolism by responding to environmental cues and shifts in nutrient homeostasis within a cell. At the core of the mTOR pathway is a serine/threonine-kinase complex that is inhibited by the mTOR agonist rapamycin (Figure 1). mTOR is made up of the two separate and functionally distinct protein complexes, mTORC1 and mTORC2. While mTORC1 functions as a protein kinase-regulating protein, controls lipid and nucleotide synthesis and prevents autophagy [2], mTORC2 is responsible for the phosphorylation of the protein kinases PKA, PKG and PKC and thus affects cytoskeletal remodeling, cell migration and proliferation [3]. mTORC2 can also modulate mTORC1 activity by promoting Protein kinase B (AKT) causing subsequent mTOR inhibition. mTORC1 lies central to the mTOR pathway and its inhibition or reduction is considered the rate-limiting step in many downstream processes. For more details see [3].

The mTOR pathway is activated by receptor tyrosine kinases (e.g., insulin receptor 1 or neurotrophic receptors) or metabotropic glutamate receptors (mGluRs) [2]. This causes the phosphorylation of the serine/threonine kinase AKT through Phosphoinositide 3-Kinase (PI3K) signaling with modulation from PTEN [4]. AKT phosphorylation causes downstream inhibition of TSC1/TSC2 (Tuberous Sclerosis Complex). TSC2 in turn can block the mTOR activator RHEB (RAS homolog enriched in brain). Activation of mTORC1 leads to phosphorylation of 4E-BP1 (eukaryotic translation initiation factor 4E-binding protein) causing decoupling of eIF4E (eukaryotic translation initiation factor 4E), which starts the translation initiation process [5]. In parallel, mTORC1 can also phosphorylate S6K1 (p70 S6 kinase 1) to propagate translation initiation, ribosomal biogenesis, and elongation [6]. 

Concurrently to receptor activation, a decrease in amino acid concentrations negatively regulates the mTOR-pathway. Specifically, GATOR1 (GAP activity towards Rags 1) negatively regulates mTORC1 activity through GTP hydrolysis of RAG A/B (Ras-related GTP-binding protein A and B) and RAG C/D (Ras-related GTP-binding protein C and D), which can also be regulated by the Ragulator complex, the lysosomal bound proteins LAMTOR 1/2/3/4/5 (lysosomal adaptor and mitogen activated protein kinase and mechanistic target of rapamycin activator) [7].

### 1.2. The RAS Pathway

Like the mTOR pathway, disruption of the RAS pathway (Figure 1) was recurrently shown to correlate with ASD severity and symptoms, cognitive impairments and intellectual disability. Several RAS pathway genes are listed among the high evidence candidates in the SFARI gene database [8,9,10]. 

At the molecular level, the RAS pathway controls cellular processes such as cell cycle and cellular growth, proliferation and differentiation, which is particularly relevant for neurodevelopmental processes [11,12]. 

The key players of the RAS pathway are members of the serine/threonine protein kinases RAS family (e.g., HRAS, KRAS and NRAS) - and RAF (e.g., ARAF, BRAF and CRAF); (Figure 1). The signaling process starts with the activation of RAS by switching from inactive GDP-bound state to active GTP-bound state. This process is tightly regulated by GTPase activating proteins (GAPs; e.g., Neurofibromin, SYNGAP1) and guanine nucleotide exchange factors (GEFs; e.g., SOS1/2). Specifically, ionotropic glutamate receptors (i.e., NMDA-R), or receptor tyrosine kinases (RTK, e.g., TrkA, TrkB) trigger this transition by activation of adaptor complexes, which include PTPN11 and by stimulation of GEFs [13]. *SYNGAP1*, one of the most frequently mutated and ASD implicated RAS genes, codes for a neuron- and Ras-specific GAP, localized at excitatory synapses, which interacts with the post-synaptic density complex and suppresses RAS signaling activation through glutamate receptor activation [14]. 

Activation of RTKs is negatively regulated by the ubiquitin ligase Casitas B-lineage Lymphoma (CBL), whereas RAF1 and BRAF activation can be blocked by SPRED1 (see review: [5]). The leucine zipper–like transcriptional regulator 1 (LZTR1) protein, an adaptor for Cullin 3 (CUL3) ubiquitin ligase complex, promotes ubiquitination of RAS, thus inhibiting its signaling functions [15,16]. 

Following activation, RAF promotes MEK1/2 phosphorylation resulting in activation of ERK1/2. Activated ERK1/2, the final effector of the signaling cascade delineated here then activates different transcription factors and regulates gene expression as well as translation. In addition to ERK, RAS proteins can modulate other signaling cascades such as mTORC1 by phosphorylating and inactivating its negative regulator TSC2. Additionally, ERK activation can mediate translation initiation also via MAPK-interacting kinase (MNK1/2), which phosphorylates eIF4E and triggers release of the FMRP-CYFIP1 complex (for an extensive review on ERK consider [17,18]).

Therefore, RAS can directly modulate downstream effects of mTOR as well as FMRP pointing to a clear coupling of these pathways, which highlights the crosstalk between signalopathies.

## 2. Clinical Phenotypes and Genetics

### 2.1. mTORopathies 

mTORopathies are defined as a co-occurring set of phenotypic symptoms, which are associated with disruptions of specific genes within the mTOR pathway, most often paralleled with ASD and epilepsy (Table 1). Most widely recognized are the Tuberous Sclerosis Complex (TSC) and PTEN Hamartoma Tumor syndrome (PTEN). New genetic studies also highlight subunits of the GATOR1 complex along with larger genetic studies pointing towards mTORC1 subunits and RHEB as being associated with a specific phenotype pattern [19,20].

There has been an increased focus on the theory of additional “second-hit” somatic mutations of mTOR-associated genes being necessary to induce ASD, specifically in developmental and epileptic encephalopathies. This also explains why a single disruptive mutation does not necessarily induce the disease phenotype. In this review we focus on the ASD phenotypes; developmental and epileptic encephalopathies are not within the scope of this article. For further reading on this topic we suggest the following reviews [21,22].

#### 2.1.1. Tuberous Sclerosis Complex

In a meta-analysis across population cohorts, the prevalence for TSC is estimated to be ~1 in 6000–10,000 with around 70% mainly associated with TSC2. Individuals with TSC have a high prevalence of epilepsy (80–90%), intellectual disability (50%) along with cortical tubers and astrocytomas [23,24]. ASD prevalence was estimated at 36% in a meta-analysis [25]. At the core of the complex are inherited (one-third of TSC individuals) or spontaneous (two-thirds) genetic mutations of the mTOR inhibitors *TSC1* and *TSC2* [26], resulting in dysregulated protein translation and an enlargement of tissues through the hyper-activation of mTOR [5]. TSC is estimated to account for ~1% of ASD patients [27]. One of the largest exome sequencing studies identified 53 *de novo* loss of function or missense mutations in a sample of 3871 ASD patients [28]. However, in comparison to the rate in controls (11.4%) this did not reach statistical significance [28].

#### 2.1.2. PTEN Hamartoma Tumor Syndrome 

PTEN hamartoma tumor syndromes cover a multitude of syndromes such as the Cowden syndrome, the Bannayan-Riley-Ruvalcaba syndrome and some cases of the Proteus and Proteus-like syndromes. In any case, the individuals carry disruptive mutations of the *PTEN* gene. Patients usually show macrocephaly, neurodevelopmental disorders, thyroid abnormalities, gastrointestinal hamartomas, mucocutaneous lesions, fibrocystic disease, breast carcinoma and uterine leiomyomas [29]. Early studies of the Cowden syndrome estimated the frequency in a Dutch population cohort at 1 in 250,000 with around 80% having *PTEN* mutations [30,31]. Individuals diagnosed with the Bannayan-Riley-Ruvalcaba syndrome have a ~60% liability for *PTEN* mutations while it is up to 50% for Proteus and up to 20% for Proteus-like patients [30,31]. The frequency of *PTEN* germ-line mutations in ASD with macrocephaly is estimated to be ~20% (for further review see [32]. Yet, in ASD cohorts *de novo*, LoF or missense variants were identified in 1% of cases with a significant difference (0.4% *q*-value < 0.05;) [28].

The PTEN-ASD profile showed reduced autism severity but with more sensory abnormalities suggestive of less sensory responsiveness. Thus, screening for *PTEN* mutations in ASD individuals, especially for those with macrocephaly and increased sensory abnormalities is suggested. 

#### 2.1.3. Additional Genetic Risk Factors of the mTOR Pathway

In addition, inactivating mutations of *RHEB* or *MTOR* result in ASD, developmental abnormalities or epilepsy resulting from mTOR hyperactivity, although their frequency is low [33,34,35]. Thorough analysis of large-scale studies will provide a clearer understanding of these less reported genes and their role amongst ASD. 

Large genetic studies of ASD cohorts report numerous *de novo* variants amongst the mTOR-related genes with the most common ASD risk-associated gene being *PTEN, TSC2* and *RAPTOR* (Table 1) [19,20,28]. It should however be noted that substantial *de novo* mutations were found related to isoforms of the eIF4e pathway, RPSK (ribosomal protein S6 kinase) pathway and PI3 kinases pointing towards mTOR dysregulation and overall abnormal translation mechanisms which could be treated with mTOR inhibitors [19]. Iossifov and colleagues report 5649 *de novo* mutations in 2500 nuclear families with ASD where again mutations of *TSC2* and *PTEN* were the highest [20]. Based on the frequencies reported as shown in Table 1 we can estimate that 0.2–0.5% of patients will carry a mutation in any of the mTOR pathway genes listed in Table 1.

Along the amino acid sensing part of the mTOR pathway lies the GATOR1 complex, which inhibits mTOR in the absence of amino acids. Mutations in two of the subunits of GATOR1 complex genes, *NPRL2* and *DEPDC5*, have been identified as major contributors of genetic focal epilepsies while often being comorbid to other diseases such as ASD (5%), intellectual disability (15%) and frontal cortical dysplasia (18%) [36]. Based upon genetic sequencing studies, *de novo* variants of *DEPDC5* or *NPRL2* may be disease causing in ~1-2 in 10,000 ASD individuals [19]). Evidence also indicates that individuals with *DEPDC5* mutations and ASD are more likely to carry an additional somatic second hit i.e., an additional mosaic mutation [37].

### 2.2. RASopathies

Somatic gain-of-function mutations of RAS genes are found in one third of human cancers [38] and germline (*de novo*) mutations are causal for several developmental disorders [39]. The best-studied RASopathies are Neurofibromatosis type 1 (NF1) and the Legius, Noonan, Costello, and Cardiofacio-Cutaneous (CFC) syndromes. All syndromes are marked by an increased prevalence of ASD and mutations within the RAS pathway (Table 1).

#### 2.2.1. Neurofibromatosis Type 1

NF1 is an autosomal dominant condition that affects about 1 in 3000 individuals worldwide and is caused by mutations of the *NF1* gene leading to hyperactivation of RAS-ERK signaling [40]. Common features of NF1 include presence of neurofibromas, bone malformations, cardiac defects and cognitive impairments (such as low IQ, attention disorder and deficits in visual-spatial skills, Table 1) (e.g., [41]). The prevalence of ASD in NF1 has recently been estimated to 10.9% [42], when correcting for recruitment biases. Interestingly, the NF1+ASD profile showed improved eye contact, less repetitive behaviors, and better language skills, with increased social-communicative impairments compared to an ASD only cohort with males and females similarly affected [43].

#### 2.2.2. Legius Syndrome

Legius syndrome is a rare autosomal dominant disorder caused by heterozygous mutations in *SPRED1*, which negatively regulates activation of BRAF and CRAF and recruits NF1 [44,45]. Legius syndrome has previously been characterized as NF1 but it is characterized by a milder phenotype compared to NF1, including freckling, macrocephaly, as well as learning impairments and attention problems [46]. Due to its similarity to NF1, the prevalence of Legius syndrome is unknown and population estimations are difficult. 

#### 2.2.3. Noonan Syndrome

Noonan syndrome occurs in approximately 1 in 2000 individuals. It is a genetically heterogeneous disorder, arising from mutations in several RAS-associated genes. Mutations of *PTPN11* account for 50% of Noonan cases, while *SOS1* is mutated in 10–13% [47,48] and *LZTR1* was found to be mutated in about 8% [49]. Other known Noonan-syndrome-associated genes include *RAF1, RIT1, KRAS, NRAS, SHOC2, BRAF, MAP2K1*, and *CBL* [50]. Individuals with Noonan syndrome are characterized by craniofacial malformations, congenital heart defects, growth and neurocognitive delay, an increased risk of developing cancer [51] with about 15% fulfilling the criteria for ASD as identified in a meta-analysis [25].

#### 2.2.4. Costello Syndrome

Costello syndrome is rare, affecting between 1 in 300,000–1,250,000 individuals and is caused by mutations of the *HRAS* gene [52]. The symptoms include delayed development, intellectual disability, cardiomyopathy, distinct facial features with curly hair, loose skin folds in the hands and feet, flexible joints, and severe feeding difficulties in infancy [53]. Two different, though small, studies report a prevalence of 26% (*N* = 44; [54]) and 44% (*N* = 11 [55]) of individuals with Costello syndrome based on the cut-off criteria for ASD of the Social Communication Questionnaire (SCQ).

#### 2.2.5. CFC Syndrome

CFCS has a prevalence of 1 in 800,000 people. It is primarily caused by *BRAF* mutations, which account for about 70% of CFC patients. Additionally, mutations in *MEK1* and *MEK2* are found in approximately 25% and mutations in *KRAS* in 2% of patients [56]. CFCS is clinically characterized by the presence of macrocephaly, ectodermal abnormalities, congenital heart defects, developmental delay as well as intellectual disability [56,57]. The SCQ cut-off for ASD was met by 54% of CFCS individuals. 

#### 2.2.6. RAS Pathway-Related Genetic Risk Factors 

In addition to the clinically defined syndromes, other single gene associated phenotypes are known: *SYNGAP1*-related intellectual disability (*SYNGAP1*-ID) is characterized by ID (100% of affected individuals), generalized epilepsy (~84%), and ASD (up to 50%) [58]. *SYNGAP1*-ID is estimated to account for at least 1% of total ID cases. Large-scale genetic studies using exome sequencing have further linked mutations in the RAS-specific, excitatory synapse located GAP *SYNGAP1* to increased risk of both ASDs [28] and schizophrenia [59]. 

Large genetic studies of ASD, epilepsy and ID cohorts have also identified *de novo* variants in RAS-related genes, among which *SYNGAP1* appears among the most significant [19,28,60] Among 5649 *de novo* mutations in a large exome sequencing study by Iossifov and colleagues [20] the mutations in *SYNGAP1* were the highest at five, followed by *LZTR1* at four and *NF1* at three individuals identified.

In a more recent study by Satterstrom et al. [19] *de novo* protein truncating and missense variants were reported as likely disease causing: i.e., in the 6430 ascertained ASD-individuals, mutations of RAS-associated genes were most frequently identified in *SYNGAP1* (ASD 14, ID 17) or *NF1* (ASD 4, ID 3). Additionally, large-scale targeted sequencing analyzing risk genes for neurodevelopmental disorders, identified *BRAF* as a gene showing a significant burden of ultra-rare (MAF < 0.01%) missense variants [61]. Like the mTOR pathway-related genes, the probability to identify a mutation in any of the RAS-associated genes is low and can be estimated to 0.2% including any of the genes listed in Table 1.

## 3. Animal Models 

To update the current view of the molecular pathomechanisms we here review rodent models of the specific signalopathies. Of note are the large proportions of published studies focusing on somatic phenotypes while only few included the behavioral or neuronal aspects. Here we focus on animal studies, which include neurodevelopmental and behavioral phenotypes related to the mTOR or RAS pathway. 

### 3.1. Animal Models of mTORopathies

#### 3.1.1. TSC

Previous reviews have summarized the extent of *Tsc1/2* heterozygous knockouts (KO) in mice [5]. To reiterate, most experiments confirm synaptic plasticity alterations, hyperexcitability in hippocampal, Purkinje and cortical neurons and seizure susceptibility [62,63,64,65]. Although survival rate of either *Tsc1* or *Tsc2* murine homozygous KO offspring is almost null, surviving pups show brain enlargement and abnormally large neurons, while *Tsc2^+/−^* heterozygous mutants show lower thresholds for late long-term potentiation [66,67]. *Tsc1^+/−^ or Tsc2^+/−^* mice show disrupted social interaction, learning and memory [66,68,69]. Dorsal telencephalon neuronal progenitor specific knockout of *Tsc1* (*Tsc1^Emx1-Cre^*) causes central nervous system malformations similar to the observed phenotypes in TSC patients [70]. More recently, conditional knockouts of *Tsc2* in mouse oligodendrocyte progenitors or in postmitotic excitatory forebrain neurons result in increased oligodendrocyte death and disruption of cortical astrocyte development, suggesting that *Tsc2* also plays a role in glial development [70,71,72].

#### 3.1.2. PTEN

A constitutive full knockout of PTEN in mice causes developmental abnormalities and is embryonically lethal. Conditional knockouts of *Pten* in hippocampal and cortical neurons or neural progenitor cells result in increased synapse density and soma size, overgrowth of dendrites and axons, augmented gliogenesis and neuronal proliferation, hypertrophy, intermittent seizure susceptibility and macrocephaly [4,73,74,75,76]. At the neurophysiological level, the same animals show increased excitatory synaptic function in hippocampal granule cells and in the basolateral amygdala [77,78]. Behaviorally, these mice exhibit anxiety like behaviors, learning impairments and ASD-related phenotypes [76]. 

Upstream of mTOR, the AKT kinases are also associated with syndromic cases as *AKT1* has been identified as a possible candidate gene for PTEN hamartoma tumor syndromes [79]. In this line, a deletion of the brain-specific AKT isoform *Akt3* leads to reduced brain size along with increased rates of dendritogenesis [80,81,82].

#### 3.1.3. mTOR Hyperactivation Models 

There is increasing evidence that the phenotypes developed from mTOR hyperactivity are a result of altered downstream protein translation. The mTOR and RAS pathways both interfere with phosphorylation and inhibition of 4E-BP and activation of S6K, respectively, two central molecules in translation initiation. Studies have shown that overexpressing the 4E-BP downstream activator EIF4E in mice leads to mTOR hyperactivation-like phenotypes such as neuroplastic abnormalities, synaptic dysfunction, repetitive behaviors, and sociability defects, all reminiscent of symptoms observed in individuals with autism [83]. Inhibition of 4E-BP recovers many of the structural phenotypes [84]. Like *Tsc1/Tsc2* heterozygous knockouts, *4e-bp* knockouts have lower thresholds for late long-term potentiation (LTP) [85]. *Rps6k* knockout mice display increased protein synthesis, cell division and smaller cell size while having deficiencies in learning and memory [86,87,88]. However, unlike *4e-bp* knockouts, *Rps6k* knockouts show no differences in late-LTP. Instead, an altered early-LTP is observed [88]. This enhanced translation is also observed in mouse models of Fragile X syndrome, a syndrome characterized by increased translational activation resulting in high incidences for ASD [89].

Knockout of *Rictor*, a component of mTORC2, results in smaller neurons and overall brain size along with increased number of neurites and decreases frequency of excitatory postsynaptic currents [90]. Full knockout of the mTOR1/2 components *Raptor* or *Rictor* results in embryonic lethality, overall decreased growth and decreased number of dendrites [80,91,92]. Deletion of mTOR in neural progenitors (*Mtor*^loxP/loxP;nestin-cre^) results in microcephaly with a reduction in progenitor replenishment leading to fewer neurons [93]. Selective chronic activation of mTOR as induced by expression of the hyperactive *mTOR* SL+IT mutant [94] in dorsal telencephalon neuronal progenitors of mice during embryonic development (*mTor^SL1+IT;Emx1-cre^)* leads to cortical atrophy with dramatic neural progenitor death while *mTor^SL1+IT;CaMKII-tTA^* mice show cortical hypertrophy, fatal epileptic seizures and aberrant recruitment of microglia [95].

Complete knockout of *Depdc5* is also embryonically lethal [96]. Heterozygous or conditional deletion of *Depdc5* in neurons results in dysplastic cortical neurons, increases cell and brain size along with alterations in cortical neuron excitability consistent with mTOR hyperactivation [37,96,97,98]. Similarly, *Depdc5* and *Nprl3* KO in mouse progenitor or neuroblastoma cells in vitro, causes increased filopodia, dendritic outgrowth and enlarged soma size [99].

Overexpression of *Rheb* WT or a mutated hyperactive *Rheb* in mice causes abnormal neuronal migration, seizures and an increased hippocampal neuronal soma size [34]. Conversely, experiments with heterozygous *Rheb* KO mice show decreased mTOR activity and result in normal synaptic plasticity, LTP and learning, suggesting that hyperactivating mutations for *Rheb* in humans are associated with mTOR hyperactivation [100]. Indeed, genetic alterations in *Mtor* propagating hyperactivation result in aberrant neuronal migration, defective neuronal ciliogenesis and enlarged cell size [35,101,102]. 

Brought together, complete knockouts of mTOR pathway genes often result in embryonic death while heterozygous or conditional knockouts produce a variety of abnormal phenotypes characterized by hyperactivation of the pathway. At the cellular level, altered cell size, increased proliferation rates, increased dendritic branching in neurons and an overall uptick in protein translation seem to be the most recurrent phenotypes. These cellular phenotypes are linked to neurodevelopmental defects characterized by cortical hypertrophy, macrocephaly and seizures, which are associated with behavioral deficits in learning, memory and social interaction and thus link mTOR hyperactivity to ASD-like behaviors.

### 3.2. Animal Models of RASopathies

#### 3.2.1. NF1

*Nf1* heterozygous knockout mice show a reduction in hippocampal spine density and attenuation of LTP [103,104]. Increased inhibitory synaptic transmission as well as alterations in presynaptic plasticity have been reported in the hippocampus, striatum and prefrontal cortex [105]. Behaviorally, *Nf1^+/−^* mice exhibit spatial and social learning deficits, reduced visuospatial attention and working memory dysfunction [106].

The conditional mouse lines with *Nf1* deletion in either inhibitory neurons (*Nf1^Dlx5/6+/−^*) or inhibitory and excitatory neurons (*Nf1^SynI+/−^)* have higher frequencies of mIPSCs (miniature inhibitory postsynaptic currents), disruption of LTP, and spatial learning deficits. In contrast, conditional heterozygous KO in neurons (*Nf1^αCaMKII+/−^*) or astrocytes (*Nf1^GFAP+/−^*) did not reveal changes in mIPSCs or LTP and did not result in learning abnormalities. These results demonstrate a critical role for neurofibromin in the regulation of GABA release from interneurons and overall mimic the symptoms (learning disabilities, visual-spatial problems) reported in NF1 patients [104,107].

#### 3.2.2. Legius Syndrome

*Spred1* knockout mice, a model for Legius syndrome, also have impaired hippocampal LTP, together with sustained LTD. The mutant animals exhibit no differences in hippocampal spine density, but show impaired hippocampus-dependent spatial memory and learning of visual discrimination, characteristics reminiscent of Legius syndrome patients [108]. Moreover, *Spred1^−/−^* mice show severe deficits in instrumental learning and social behavior, such as enhanced social dominance, altered ultrasonic vocalization and nesting behavior [109,110]. Thus, many of the molecular mechanisms of *Spred1* knockout mice remain elusive and still need to be addressed. 

#### 3.2.3. Noonan Syndrome

The most used mouse model to study Noonan syndrome is the *Ptpn11* knock-in model. *Ptpn11^D61G/+^* mice show strong LTP and spatial learning deficits, whereas *Ptpn11^N308D/+^* mice exhibit milder effects, reflecting patients carrying the respective mutations [111]. *Ptpn11^D61G/+^* mice have increased AMPAR levels and enhanced excitatory synaptic transmission, while dendritic spine density has not been investigated. Neuron-specific conditional knockout mice, *Ptpn11^αCaMKII-cre^* show a milder phenotype including minor delays in spatial learning and increased hyperactivity with no changes in LTP [112]. *Ptpn11^E76K/+;Nestin-Cre^* mice develop a hydrocephalus and exhibit hyperactivity paralleled by reduced anxiety behavior [113]. 

Other Noonan mouse models are the *Raf1^L613V^* heterozygous mice, which show an atypically high density of astrocytes and oligodendrocyte progenitors in the cortex and hippocampus [114]. Interestingly, *Raf1*^L613V^ mice show enhanced performance in several learning tasks. 

#### 3.2.4. CFC Syndrome

Homozygous deletion of *Braf* is lethal. The *Braf* knock-in model *Braf ^Q241R/+^* is based on the most common CFC mutation identified in patients. It has primarily been investigated for its cardiac phenotype and impairments in contextual fear learning [115,116]. While effects on synaptic plasticity have not been reported, *Braf^V600E/+^* shows a strong epileptic phenotype, which is a common characteristic of CFC patients with *BRAF* mutations [117,118]. Neuron-specific conditional (*Braf^αCAMKII-cre^*) knockout mice have normal basal synaptic transmission but impaired maintenance of early-phase LTP, poor acquisition in the spatial learning task, significantly impaired spatial memory and context discrimination [119]. 

Other CFC models include those with *Kras* and *Mek1* mutations. The *Kras^G12V/+;Syn1-cre^* model exhibits reduced hippocampal LTP and increased inhibitory tone throughout the brain, possibly due to increased ERK activity during postnatal development. Behaviorally, these mice show various deficits in spatial and working memory but have normal social behaviors [120]. Whereas the dnMEK1 (inhibiting ERK) mouse model shows typical behavioral impairments in spatial learning, long-term memory and fear conditioning, *Mek1^Y130^* has not been characterized in this context, but shows other CFC symptoms such as cranial dysmorphia and neurological anomalies, including increased astroglial density in the cortex [121,122]. Hyperactive MEK1 signaling in cortical GABAergic neurons of *Mek1^Slc32A1:Cre^* mice promotes embryonic parvalbumin neuron loss and shows defects in behavioral response inhibition [123].

#### 3.2.5. Costello Syndrome

*Hras^G12V/G12V^* homozygous knock-in mice show enhanced anxiety and mild cognitive deficits in spatial learning but normal interneuron excitability [124]. Mice with the *Hras^G12V^* mutation under the control of the Synapsin promoter have an increased size of pyramidal neurons as well as increased complexity and spine density of dendritic trees [125]. Additionally, *Hras*^G12V^ mice show increased production of cortical neurons, increased soma size and decreased total neurite length, whereas mice with an astrocyte-specific *Hras* mutation exhibit impaired cortical maturation during development and abnormal extracellular matrix remodeling [126,127]. Interestingly, *Hras^G12VαCaMKII^* mice have enhanced LTP revealing a presynaptic role of HRAS, as deleting Synapsin I in *Hras^G12VαCaMKII^* mice prevented this enhancement [128]. This finding was similarly reported in Costello syndrome patients, although the patients have cognitive deficits. However, in *Hras^G12VαCaMKII^* mice the enhancement of LTP was mirrored by the enhanced hippocampus-dependent learning in the MWM and in contextual fear conditioning [128]. 

#### 3.2.6. SYNGAP1

Given that Syngap1 is a key signaling component in LTP and regulates spine enlargement upon LTP, it is not surprising that *Syngap1^+/−^* mice have a reduced magnitude of LTP and early spine maturation [129]. In addition, *Syngap1^+/−^* mice show enhanced mGluR-LTD, elevated basal protein synthesis and deficits in excitation/inhibition balance in the cortex [130,131]. Reducing SYNGAP1 expression in adulthood has minimal impact on the AMPA/NMDA ratio and cognition, demonstrating a crucial role for SYNGAP1 in neurodevelopment [132]. 

*Syngap1^+/−^* mice exhibit a number of behavioral phenotypes including increased locomotor activity, decreased anxiety-like behavior, impaired spatial and working memory as well as increased stereotypic behavior, reduced social novelty preference, and impaired cued fear memory [133,134]. Importantly, haploinsufficiency in GABAergic inhibitory neurons does not impair cognition or neurotransmission whereas heterozygous knockout in excitatory neurons leads to the reported behavioral phenotypes [135].

Taken together, mouse models of RASopathies, although genetically diverse, converge on specific phenotypic traits. At the cellular level these commonly include enlarged cell bodies, increased astroglial and reduced neuronal cell densities, and deficits in dendritic spine maturation and density. Physiologically, impairments in synaptic transmission are observed, such as decreased hippocampal LTP, and excitatory-inhibitory imbalance. Behaviorally, these models show various learning and memory deficits, hyperactivity and social interaction defects.

In summary, alterations of RAS or mTOR signaling at the genetic and molecular level converge at the level of synaptic plasticity specifically leading to an altered excitatory/inhibitory imbalance and memory deficits. Additionally, some models display ASD-like phenotypes and neurodevelopmental abnormalities. 

This is in line with the observation that disruptive mutations of mTOR and/or RAS-signaling associated genes are more prominent in ASD individuals with low cognitive performance and morphological abnormalities. However, a deep behavioral characterization and rescue experiments in animal models at the behavioral level are often lacking. Overall, we conclude that the animal models are particularly well suited to dissect the pathomechanisms at the functional level. This can and should be paralleled by human cell models as these models allow investigating human specific functional patterns or developmental trajectories, along with the possibility to study patient-specific genetic backgrounds. 

## 4. Human Cell Models

The recent progression in the use of human stem cell models, specifically the reprogramming, gene-editing and targeted differentiation of patient cells into neuronal models in 2D and 3D allow getting a more human-oriented understanding of the pathomechanisms underlying signalopathies. Here, we specifically aimed at reviewing recent human neuronal cell-model phenotypes and their comparisons to rodent models (Table 1). Neurons derived from embryonic stem cells (ESCs) or induced pluripotent stem cells (iPSCs) are currently most widely used to study human neuronal differentiation *in vitro*. Both types of stem cells can be differentiated into neural progenitor populations, which subsequently can be differentiated into neurons for functional analyses. To investigate a certain gene of interest, iPSCs are either directly generated from patients or genetically edited e.g., using CRISPR/Cas9 to mimic or repair gene disruptions occurring in patients. 

Varying methods for neuronal differentiation have been developed, including the direct neuronal conversion of either fibroblasts or stem cells (iNeurons) driven by the forced expression of transcription factors, which, in contrast to differentiation via respective growth factors alone, results in almost pure neuronal cultures [136,137]. Lastly, 3D models, e.g., cerebral organoids or “mini-brains” generated from human stem cells provide a more in vivo-like framework for understanding developmental aspects characteristic of humans, allowing to at least partially model cortical layer formation and brain folding, a structural feature absent in mice or rats.

### 4.1. Cell Models of mTORopathies 

#### 4.1.1. TSC 

A few comprehensive overviews of human cell models with *TSC1* and *TSC2* knockouts in iPSCs, ESCs, and 3D organoid cultures were published recently [138,139]. Most studies are based on patient-specific iPSCs generated from *TSC1* or *TSC2* mutation carriers and indicate an overall decrease in neurons comparative to their respective controls [140,141,142,143]. However, it is uncertain whether this is due to an increase in astroglia, delayed neuronal differentiation or increased neuronal death [63,72,144,145]. Interestingly, cell lines with heterozygous *TSC1/2* mutations show reduced abnormalities compared to homozygous knockouts suggesting biallelic knockouts to be necessary to fully develop the TSC phenotype including cortical tubers. Thus, giving evidence to the possibility of a “two-hit” model where heterozygous mutations require a second mutation in the corresponding allele to generate the disease phenotype.

Transcriptome analysis of *TSC2*-deficient heterozygous neural stem cells (NSCs) derived from ESCs or iPSCs has also confirmed increased protein synthesis, metabolic activity, gliogenesis and inflammatory responses while having decreased transcriptomic signatures for neuronal differentiation, reinforcing previous observations in human and animal models [66,70,143,146]. 

Recent research in a *TSC2* knockout iPSC model confirms the altered excitatory/inhibitory imbalance as observed in animal models, which is attributed to an increase in GABAergic signaling at inhibitory synapses [147]. However, neuronal growth cone studies in neurons derived from iPSCs report *TSC2* heterozygous knockouts to have normal mTOR signaling but abnormal axon extension and insensitivity to inhibitory axonal guidance cues suggesting mTOR downstream processes to be the culprit and not necessarily mTOR itself [148]. 

Investigations in cerebral organoids of *TSC2* patients with drug resistant epilepsy and cortical tuber development have shown mTOR hyperactivity, increased proliferation and subsequent generation of cortical tubers in vitro specifically in a caudal late interneuron progenitor population. Single-cell sequencing suggests an upregulation of EGFR being causal for the effects which were rescued by drug treatments targeting mTOR or EGFR itself [149]. 

#### 4.1.2. PTEN

*PTEN* has been explored in cell models for its widely heterogenous function including its role as a tumor suppressor gene with only few studies focusing on neuronal phenotypes. Investigations in human models conclude that more severe phenotypes have an underlying decreased protein half-life based on increased polyubiquitination [150,151]. Specifically relevant for ASD are the observations of an increased number of neural progenitors and dendritic outgrowth associated with PTEN knock-in and knockout, which might underlie the aberrant neuronal development observed in patients’ brain structures. PTEN is instrumental during the development of dopaminergic neurons by cross talking to ERK signaling as shown in human NSCs [152]. In an organoid model generated from a human ESC CRISPR/Cas9-induced *PTEN* mutation Li and colleagues confirmed a delayed neuronal differentiation with an overall increased organoid size compared to controls [153]. Interestingly, when compared to mouse organoids, human ESC *PTEN* knockout organoids produced higher surface folding, clearly indicating species differences in development, and thus pointing towards the necessity of human neuronal models in understanding the role of PTEN in brain structure. 

#### 4.1.3. DEPDC5

Klofas et al. differentiated neurons from iPSCs generated from patients with heterozygous *DEPDC5* mutations. Neural progenitor cells show increased mTOR activity and increased proliferation rates while simultaneously enlarging soma size. Neurons are also observed to have increased soma size indicative of mTOR hyperactivation [154]. 

Despite extensive animal studies, there seems to be a lack of strong human cellular based systems for other mTOR pathway disease susceptible genes relating to brain development. Studies of other previously mentioned mTOR related genes have focused on other organs or systems than the brain emphasizing the need to generate and confirm the effects of knockout genes and their pathology amongst human cellular models. The available technologies and methods using ESC- and iPSC-derived human neuronal models can and need to be utilized for targeted drug development and validation to reduce the risk for unforeseen differences between species such as seen in organoids with *PTEN* mutations [153]. 

### 4.2. Cell Models of RASopathies 

#### 4.2.1. Neurofibromatosis Type 1

Several iPSC lines either reprogrammed from skin fibroblasts or from plexiform neurofibroma cells have been used to study NF1 in vitro (also see review [155]). 

iPSC-derived neural progenitor cells (NPCs) of patients with distinct *NF1*(+/−) mutations confirm that regulation of cyclic AMP (cAMP) through neurofibromin (NF1 protein) is dependent on the activation of RAS [156]. Interestingly, all *NF1* patient-derived NPCs show increased RAS activation and reduced cAMP production, respectively. Furthermore, a neurofibromin dose-dependent reduction in dopamine levels has been described for all *NF1* deficient cell lines [157]. 

To study specific *NF1* mutations, 7 patient-derived iPSC lines were differentiated in 2D and 3D neuronal models [158]. While in 2D models all NPC-lines as well as astrocytes show an increased proliferation and RAS activity, distinct effects on dopamine levels are observed in NPC lines in specific mutations only. In the respective 3D cerebral organoids, the authors observe striking differences between the mutations with respect to rates of NPC proliferation, apoptosis, and neuronal differentiation, suggesting that the clinical heterogeneity of NF1 syndrome might originate from the distinct *NF1* mutations, or the difference in their genetic background [158].

iNeurons of heterozygous *NF1* mutation carriers show decreased expression of mex-3 RNA binding family member D (MEX3D), accompanied by a higher expression of FOS after two weeks of differentiation [159]. In contrast, mRNA levels of the apoptosis regulator B-cell lymphoma 2 are increased during early stages of neuronal differentiation (5 days), while there is no difference between control and *NF1*-iNeurons after two weeks. Thus, this study points towards a dysregulation of apoptosis during neuronal differentiation of *NF1*-iNeurons, a pathomechanism previously described in the etiology of ASD [160]. The fact that mRNA levels of *Mex3d* are not affected by *Nf1* knockdown in mouse Neuro2A cells [159] furthermore underlines the need and translatability of human cell model systems.

In a follow-up pilot study, however, comparing one *NF1*-iNeuron to one control-iNeuron line, the authors could reverse the observed effects via forskolin (increasing cAMP expression via stimulation of adenylate cyclase) administration during differentiation [161]. These findings will need further validation with higher sample sizes. 

#### 4.2.2. Noonan Syndrome

Although iPSC lines are a valuable resource for neurodevelopmental disease research, so far most of the cell lines generated for the various genes associated with Noonan syndrome have only been studied in the context of the Noonan-associated cardiomyopathy (*RAF1* [162,163], *LZTR1* [164], *MRAS* [165]) and juvenile myelomonocytic leukemia (*PTPN11* [166]). Only one study aimed at analyzing neurodevelopment using iPSCs from three patients diagnosed with Noonan syndrome (NS-iPSCs) harboring mutations in *PTPN11* [166]. Interestingly, embryoid bodies (EBs) generated from NS-iPSCs are not able to differentiate into neuroectoderm, which is driven by an activation of BMP and TGF-beta signaling. However, inhibition of either pathway rescues the observed effects and is used to push the differentiation towards neuronal cells in 2D and 3D. The generated NS-neural cell lines both show increased gliogenesis as well as shortened neurites accompanied by lower spontaneous firing rates, respectively. These phenotypes are rescued via inhibition of PTPN11 indicating a role in the establishment of electrophysiological properties in NS-neural cells via an imbalanced population of neurons and glial cells as well as reduced neurite outgrowth [166]. 

#### 4.2.3. CFC Syndrome

At the time writing, only two studies focused on human neurodevelopmental aspects of CFCS in vitro. 

Two iPSC lines generated from a CFCS patient with a mutation in *BRAF* (gain-of-function, p.Q257R, c.770A>G) fail to differentiate into neurospheres due to an enhanced activation of SMAD1 and ERK signaling accompanied by beta-catenin delocalization [167]. Inhibition of SMAD1 does not only rescue EB morphology but restores neuronal differentiation and beta-catenin localization. This indicates that disturbed SMAD1 signaling during early development is a major pathomechanism underlying the aetiology of CFCS. 

In contrast, the other study also investigating iPSC-derived neurons of four patients with the same p.Q257R gain-of-function *BRAF* mutation shows premature differentiation and an imbalance of neural cell types, i.e., an increase in deep-layer cortical neurons and a depletion of upper-layer cortical neurons [168]. The observed effects are rescued by the activation of PI3K/AKT signaling.

Interestingly, these two mentioned studies differ in their differentiation methods in that the latter uses SMAD inhibition as a driver for neuronal differentiation while the first one utilizes the withdrawal of growth factors. This highlights the importance of the chosen differentiation method as this choice has the potential to mask certain phenotypes.

#### 4.2.4. Costello Syndrome

iPSCs of patients with heterozygous *HRAS^G12S^* mutations in comparison to those of typically developing controls differentiate faster into astroglia, show hyperplasia, and express higher levels of extracellular matrix remodeling factors and proteoglycans, as well as a dysregulated cortical maturation during development [127]. The increased expression of proteoglycans is reversed by administration of farnesyl transferase inhibitor as well as by a knockdown of the transcription factor *SNAI2*, which is known to be regulated by RAS signaling. 

In another study investigating the role of aberrant RAS signaling in human neuronal differentiation, neuroectodermal differentiation of iPSCs generated from patients with Costello syndrome results in an extended progenitor phase with a subsequently increased production of cortical neurons [126]. Mature neurons differentiated from patient iPSCs show decreased neurite length and increased soma size.

iPSC lines are a valuable resource for studying neurodevelopment although in modelling RASopathies they so far were mostly studied in the context of other symptoms of the syndrome, such as cardiomyopathy or leukemia. The so far available studies on neurodevelopment have shown dysregulated proliferation and differentiation, reduced neurite outgrowth, imbalanced population of neurons and glial cells, and lower spontaneous firing rates. However, the phenotypes observed are often strikingly different, suggesting that these might originate from the distinct gene mutations or the difference in the genetic background thus emphasizing the need for optimized and unified protocols when comparing the outcomes.

## 5. Pharmacological Interventions 

### 5.1. mTORopathies 

Being a master-regulator, the mTOR pathway moved into the focus of pharmaceutical industry many years ago, where it is considered a potent multi-valued drug target. The pioneering compound rapamycin, an effective inhibitor of mTORC1, has been studied extensively for its antitumor properties. In 2010, the rapamycin analog Everolimus, a so-called “rapalog”, was approved as treatment of subependymal giant cell astrocytomas in TSC patients [169]. Given the growing body of genetic and molecular evidence for mTORC1 involvement in neurodevelopmental disorders, there has been increased interest in compounds such as Sirolimus (rapamycin) and Everolimus as potential treatments for symptoms of mTORopathies. Based on the numerous studies proving the efficacy of rapamycin in suppressing seizures in animal models of mTOR hyperactivity [170] Everolimus has been approved as treatment for epilepsy in TSC in 2018. Besides its anti-epileptic effect, rapamycin has been shown to reverse sociability defects in a TSC mouse model [68]. However, until today there are no clinical studies proving that mTOR inhibition ameliorates autism symptoms or intellectual disability in mTORopathies in human. For example, a randomized clinical trial completed in 2018 has shown that treatment of 6- to 21-year-old TSC patients with 4.5 mg of Everolimus daily over 6 months has no significant effects on the level of their memory, sociability or sleep behavior (ClinicalTrials.gov Identifier: NCT01289912). Yet, it remains a possibility that other forms of mTORopathies respond differently. For example, a Phase I/II 6 months clinical trial evaluating the potential neurocognitive benefits of treatment with Everolimus in a patient population with *PTEN* mutations is still ongoing (NCT02991807).

On the other hand, the negative outcome [171] of Everolimus treatments in the context of autism might not be a matter of inefficacy but a question of critical developmental windows. Indeed, several studies in mouse models of TSC reveal the potential of early postnatal rapamycin administration to reverse histopathological phenotypes of mTOR hyperactivation [172,173]. These findings indicate that earlier treatment intervention with rapalogs could potentially ameliorate cognitive defects such as autism symptoms in patients. Along this line, a current Phase I/II clinical trial (STOP2 - NCT04595513) assesses the effects of Everolimus on epilepsy prevention in TSC infants up to 6 months of age. This study will elucidate if pharmacological mTOR inhibition during critical neurodevelopmental time windows alleviates cognitive impairments in mTORopathies. Thereof, there is ongoing effort to develop more stable rapalogs with better brain permeability and less systemic toxicity [174,175,176]. 

Besides pharmacological inhibition of mTOR signaling, other treatment avenues for mTORopathies involve for example the administration of anticonvulsants such as Vigabatrin during critical developmental time windows. An ongoing Phase II clinical trial investigates if low doses of Vigabatrin provided to newborns for up to 24 months, can prevent epilepsy and cognitive defects in TSC patients (NCT02849457). Further, a direct pharmacological inhibition of Akt in the context of *Pten* mutations has shown promising results *in vitro*, thereby accentuating a certain amenability to other pharmacological treatments than “rapalogs” [177].

The current state of research shows that modulation of mTOR activity allows us to successfully treat comorbidities of mTORopathies such as epilepsy. However, treatment interventions improving cognitive phenotypes of mTOR hyperactivation remain to be identified. This can partially be attributed to the fact that traditional drug screens have been performed on cancer cell lines or transgenic animals, which do not recapitulate a patient’s unique genetic background. Now, with the advent of human iPSC technology, patient-derived cells can be used to study disease-associated phenotypes in a subject- and cell-type specific manner [178]. In addition, their differentiated derivatives can further be employed for high-throughput screens for disease modulating compounds, thereby paving the way for precision medicine in the context of mTORopathies.

### 5.2. RASopathies

Many of the morphological and behavioral phenotypes of human RASopathies can be successfully phenocopied in model organisms allowing these models for testing various drugs [179]. Specifically, statins (3-hydroxy-3-methylglutaryl coenzyme A reductase inhibitors), which are commonly used to treat hypercholesterolemia and were tried as cancer therapeutics, have been tested in treating RASopathies. In mouse models for NF1 (*Nf1^+/−^ mice*), Noonan syndrome (*Ptpn11^D61G/+^ mice*) and SYNGAP1-ID (*Syngap1^+/−^* mice) lovastatin successfully ameliorates the cognitive deficits, restoring normal protein synthesis, improving memory and spatial learning [111,130,180]. Lamotrigine, an antiepileptic drug and HCN channel agonist, rescues the electrophysiological and cognitive deficits in *Nf1^+/−^* mice [104]. The outcomes of clinical trials, however, show high variability, with some demonstrating potential benefits of lovastatin and simvastatin, and others failing to prove their efficacy [181,182,183,184,185,186]. Currently, it is being tested whether lovastatin or lamotrigine can improve synaptic plasticity and cognitive function in Noonan syndrome and NF1 patients (NCT03504501). The efficacy of lovastatin is being assessed on reading disability of NF1 patients (NCT02964884). A recently closed but not yet evaluated study aimed at investigating synaptic physiology and behavioral inhibition in patients with NF1 and ASD and to answer whether inhibitory deficits at these levels are modulated by lovastatin (NCT03826940). 

MEK inhibitors are mostly used to target tumors or malignancies within RASopathies. In various mouse models (e.g., *Sos1, Raf1, Kras*) treatment with PD0325901 (Mirdametinib) resulted in increased survival, improved stature, and craniofacial features [187,188]. In adult *Spred1^+/−^* mice acute PD325901 treatment does not reverse severe instrumental learning deficits, but is successful in reversing social dominance and improving nesting behavior [109,110]. The MEK inhibitor Selumetinib has been approved for treating large plexiform neurofibromas in NF1 patients [189]. BRAF and MEK inhibitors, Dabrafenib and Trametinib, have been approved for the treatment of BRAF V600E/K-mutant melanoma but only show transient clinical benefit due to the rapid onset of resistance. Therefore, ERK inhibitors such as Ulixertinib are being considered [190] as treatment for cancers harboring both *BRAF* and *NRAS* mutations. Aside from cancer treatments, Trametinib is being tested as a treatment for hypertrophic cardiomyopathy in Noonan syndrome patients [191]. Interestingly, ERK inhibitor treatment rescues the molecular, anatomical and behavioral deficits of *16p11.2* (one of the most common gene copy number variations linked to autism) deletion in mice [192], and newly developed ERK pathway inhibitor peptides ameliorate defective synaptogenesis in the *Kras^12V^* mouse model [120]. However, at the time writing, no clinical trial has investigated the benefits at behavioral level. 

Aside from directly targeting the RAS-pathway, a different approach has been discussed, which focuses on modulatory or “accessory proteins” as new potential therapeutic targets [193]. The main idea is not to completely abolish this essential pathway, but to modulate it and reduce it to physiological levels. For example, genetic inhibition of *SHOC2* suppresses tumor development and prolongs overall survival in KRAS-driven lung adenocarcinoma mouse models. In addition, it inhibits tumorigenic growth in a subset of KRAS- and EGFR-mutant human cell lines [194]. Importantly, *SHOC2* deletion is well tolerated, both in human cell model and in adult mice, suggesting a therapeutic strategy to treat RAS- and EGFR-mutant cancers. 

## 6. Conclusions

The review of the molecular, cellular and physiological studies in rodent and human models converges on a disruption of the RAS and/or the mTOR pathway being associated with the observed neurodevelopmental phenotypes. Specifically, macro- or microcephaly, ADHD comorbidity and/or ID including ASD are among the best replicated observations. 

The overall proportion of ASD cases which can be explained by a signalopathy is low. However, the estimations in the ASD cohort are likely to be biased by the low total number of mutations reported as well as by the study designs, i.e., the exclusion of patients with known genetic disorders. The presence of disrupting variants in typically developing controls further suggests a multiple-hit model. Finally, ASD-specific symptom severity often does not seem to correlate with mutation burden in the RAS or mTOR pathways.

However, the probability to identify a signalopathy-related mutation in an ASD cohort is 2 to 20 times higher than in the general population, depending on the preselection of the ASD individuals (e.g., with macrocephaly). Thus, specifically in individuals with marked phenotypic characteristics, targeted exome sequencing screening for disruptive variants helps identifying patients with aberrant RAS or mTOR signaling. Given recent developments and efforts in drug development, chances are high that more specific treatment options will be available for ASD cases with an identified signalopathy. Currently, treatments of signalopathies at the neuronal level are available for NF1 and for ASD with comorbid epilepsy as well as brain cancers. Thus, the identification of signalopathies is essential to inform treatment and specifically, to indicate potential somatic comorbidities (e.g., cancers), which would not have been identified in a psychiatric setting.

Although animal and cellular models of disease-susceptible genes can provide a clear avenue for therapeutic targets, identifying the treatment time window remains specifically challenging, i.e., is there a specific time-period during brain development when treatment can reverse, reduce, or prevent a specific phenotype. Hence, we need a better understanding of the role of the RAS and mTOR pathway during neurodevelopment in order to identify the mechanisms modulating or preventing aberrant regulation in typical controls. 

To foster clinical translatability and to leverage the clinical benefit we further suggest aiming at improving our understanding of the differential roles of the signaling pathways between human and animal models, and to test the efficiency of drugs in animal models and human cell models in order to guide preferentially targeted drug selection. 

## Figures and Tables

**Table 1:** Summary of selected signalopathy-associated clinical phenotypes and respective findings in rodent and human cell models.

	Clinical Phenotypes (Frequency)	Animal Models	Human Cell Models	Mutations in 10,000 ASD Cases [19]
**mTOR Pathway**				
*TSC1-2*	**Tuberous Sclerosis (1:10,000)** ASD, ID, epilepsy, structural neurological changes, cancers/tumors, skin problems	Altered synaptic plasticity; altered social behavior; altered learning behavior and memory; disrupted neuronal cell development; seizures	Increased dendritic branching; altered synaptic plasticity; enlarged cell size	*TSC1*: n.d. *TSC2*: 3.8
*PTEN*	**Cowden Syndrome** **(1:250,000)** ASD, ID, epilepsy, structural neurological changes, cancers/tumors, skin problems	Altered synaptic plasticity; altered social behavior; altered learning behavior and memory; disrupted neuronal cell development; seizures	Impaired cortical folding	*PTEN*: 4.4
*AKT1-3*	**Cowden Syndrome** **(1–9:1,000,000) and** **AKT1-related Proteus Syndrome** ASD, ID, epilepsy, structural neurological changes, cancers/tumors	Altered synaptic plasticity; altered social behavior; altered learning behavior and memory; disrupted neuronal cell development; seizures	Increased dendritic branching; altered synaptic plasticity; enlarged cell size	*AKT1*: n.d. *AKT2*: 1.3 *AKT3*: 1.3
*MTOR*	ASD, ID, epilepsy, structural neurological changes, cancers/tumors	Disrupted neuronal cell development; seizures		*MTOR*: 0.6
*RICTOR/RAPTOR*	ASD, ID, epilepsy, structural neurological changes, cancers/tumors	Altered synaptic plasticity; disrupted neuronal cell development		*RICTOR*: n.d. *RAPTOR*: 2.5
*RHEB*	ASD, ID, epilepsy, structural neurological changes, cancers/tumors	Altered synaptic plasticity; disrupted neuronal cell development; seizures		n.d.
*S6K*	ASD, ID, epilepsy, structural neurological changes, cancers/tumors, skin problems	Minor altered synaptic plasticity; altered learning behavior and memory; altered social behavior; disrupted neuronal cell development		*RPS6KB1*: 1.9
*eIF4EeIF4E-B*	ASD, ID, epilepsy, structural neurological changes, cancers/tumors	Altered synaptic plasticity; altered social behavior; altered learning behavior and memory; disrupted neuronal cell development		*EIF4B*: 0.6
*DEPDC5*	ASD, ID, epilepsy, structural neurological changes, cancers/tumors	Altered synaptic plasticity; disrupted neuronal cell development; seizures	Enlarged cell size; increased proliferation rates	*DEPDC5*: 0.6
*NPRL2*	ASD, ID, epilepsy, structural neurological changes, cancers/tumors			n.d
**RAS Pathway**				
*SYNGAP1*	**SYNGAP1-ID** ASD, ID, epilepsy, schizophrenia	Altered synaptic plasticity (reduced hippocampal LTP and early spine maturation); elevated protein synthesis; altered spatial learning and memory, social novelty, fear memory and increased locomotor activity		*SYNGAP1*: 10.8
*NF1*	**NF1 Syndrome (1: 3000)**: ASD, ID, structural neurological changes, congenital heart disease, bone malformations, cancers/tumors, skin problems	Altered synaptic plasticity (reduced hippocampal LTP and spine density); altered spatial learning and memory, social learning, attention and working memory deficits	Altered proliferation, apoptosis and neuronal differentiation	*NF1*: 4.4
*SPRED1*	**Legius Syndrome** ASD, ID, structural neurological changes., congenital heart disease, skin problems	Altered synaptic plasticity (reduced hippocampal LTP), altered spatial learning and memory and social behavior		*SPRED1*: 0.6
*SOS1*	**Noonan Syndrome (1:2000)** ASD, ID, epilepsy, structural neurological changes, congenital heart disease, bone malformations, cancers/tumors	craniofacial abnormalities; cardiac defects		n.d.
*CBL*	**Noonan Syndrome (1:2000)** ASD, ID, epilepsy, structural neurological changes, congenital heart disease, bone malformations, cancers/tumors			*CBL*: 0.6
*HRAS*	**Costello Syndrome (1:300,000–1.25 mil.)** ASD, ID, structural neurological changes, congenital heart disease, bone malformations, cancers/tumors, skin problems	Increased soma size and spine complexity: enhanced LTP; altered spatial learning and memory and contextual fear conditioning	Increased astrogenesis, increased proteoglycans, dysregulated cortical maturation	n.d.
*NRAS*	**Noonan Syndrome (1:2000)** ASD, ID, epilepsy, structural neurological changes, congenital heart disease, bone malformations, cancers/tumors	Leukemias, craniofacial abnormalities, cardiac defects		n.d.
*KRAS*	**CFC and Noonan Syndrome** ASD, ID, epilepsy, structural neurological changes, congenital heart disease, bone malformations, cancers/tumors	Altered synaptic plasticity (reduced hippocampal LTP); elevated protein synthesis; altered spatial learning and memory, working memory; normal social behaviors		*KRAS*: 0.6
*RIT1*	**Noonan Syndrome (1:2000)** ASD, ID, epilepsy, structural neurological changes, congenital heart disease, bone malformations, cancers/tumors	Reduced dendritic length and complexity		n.d.
*RAF1*	**Noonan Syndrome (1:2000)** ASD, ID, epilepsy, structural neurological changes, congenital heart disease, bone malformations, cancers/tumors	Increased astroglial cell density; enhanced spatial learning and memory		n.d.
*BRAF*	**CFC and Noonan Syndrome** ASD, ID, epilepsy, structural neurological changes, congenital heart disease, bone malformations, cancers/tumors	Altered synaptic plasticity (reduced hippocampal LTP); altered spatial learning and memory; seizures	Inhibited neuronal differentiation; premature differentiation and impaired cortical layering	*BRAF*: 1.3
*MEK1/2*	**CFC Syndrome (1:800,000)** ASD, ID, epilepsy, structural neurological changes, congenital heart disease, bone malformations, cancers/tumors	Increased astroglial cells, neuronal loss; altered spatial learning and memory; altered fear conditioning; craniofacial abnormalities		*MAP2K1*: 0.6*MAP2K2*: 1.3
*PTPN11 (SHP2)*	**Noonan Syndrome (1:2000)** ASD, ID, epilepsy, structural neurological changes, congenital heart disease, bone malformations, cancers/tumors	Altered synaptic plasticity; altered spatial learning and memory; increased hyperactivity, reduced anxiety behavior	Inhibited differentiation, increased gliogenesis; reduced neurite outgrowth; lower spontaneous firing rate	*PTPN11*: 1.3
*LZTR1*	**Noonan Syndrome (1:2000)**			*LZTR1*: 3.2

Abbreviations: ASD Autism Spectrum Disorder, ID Intellectual Disability, LTP Long Term Potentiation; n.d. not detected. The central disorders are in bold.

## Abbreviations

AMPAR	α-amino-3-hydroxy-5-methyl-4-isoxazolepropionic acid receptor
ASD	Autism Spectrum Disorder;
AKT	protein kinase B;
BRAF	V-Raf murine sarcoma viral oncogene homolog B;
Camp	cyclic adenosine monophosphate;
CBL	Casitas B-lineage Lymphoma;
CFCS	Cardiofacio-Cutaneous syndrome;
CUL3	Cullin 3;
DEPDC5	DEP domain containing 5 protein;
EB	embryoid bodies;
eIF4E	eukaryotic translation initiation factor 4E;
EGFR	epidermal growth factor receptor;
ERK 1/2	extracellular signal-regulated kinase 1/2;
ESC	embryonic stem cells;
GABA	gamma-aminobutyric acid;
GAP	GTPase activating protein;
GATOR1	GAP activity towards Rags 1;
GEF	guanine nucleotide exchange factor;
HCN	hyperpolarization activated cyclic nucleotide gated potassium and sodium channel;
ID	Intellectual Disability;
iPSC	induced pluripotent stem cells;
iNeurons	induced Neurons;
KO	knockout;
KRAS	kirsten rat sarcoma virus proto-oncogene, GTPase;
LAMTOR	also known as Ragulator, late endosomal/lysosomal adaptor and mitogen activated protein kinase and mechanistic target of rapamycin activator;
LTP	Long-term potentiation;
LTD	Long-term depression;
LZTR1	leucine zipper–like transcriptional regulator 1;
MAF	minor allele frequency;
MAP2K1/2 or MEK 1/2	mitogen activated protein kinase 1/2;
MEX3D	mex-3 RNA binding family member D;
MGLURs	metabotropic glutamate receptors;
MNK1/2	MAPK-interacting kinase;
MTORC1/2	mammalian target of rapamycin complex 1/2;
MWM	Morris water maze;
NCT	National Clinical Trial number
NF1	Neurofibromatosis type 1;
NMDA-R	N-methyl-D-aspartic acid receptor;
NPC	neural progenitor cell;
NPRL2	nitrogen permease regulator 2-like protein;
NRAS	neuroblastoma RAS viral oncogene homolog;
NS	Noonan Syndrome;
NSC	neural stem cell;
PI3K	Phosphoinositide 3-Kinase;
PTEN	Phosphatase and Tensin homolog;
RAF1	Raf-1 proto-oncogene, Serine/threonine kinase;
Rag A/B, C/D	Ras-related GTP-binding protein A/B, C/D;
Raptor	regulatory-associated protein of mTOR
RHEB	Ras homolog enriched in brain;
Rictor	rapamycin-insensitive companion of mTOR;
RIT1	Ras like without CAAX1;
RPSK	ribosomal protein S6 kinase;
RTK	receptor tyrosine kinases;
SHOC2	SHOC2 leucine rich repeat scaffold protein 2;
SMAD1	mothers against decapentaplegic homolog 1;
SPRED1	sprouty-related, EVH1 domain-containing protein 1
SYNGAP1	synaptic Ras GTPase Activating Protein 1;
SYNGAP1-ID	SYNGAP1-related intellectual disability;
S6K1	p70 S6 kinase 1;
TSC	Tuberous Sclerosis Complex;
4E-BP1	eukaryotic translation initiation factor 4E-binding protein.
